# 24-Epibrassinolide alleviates diazinon oxidative damage by escalating activities of antioxidant defense systems in maize plants

**DOI:** 10.1038/s41598-023-46764-y

**Published:** 2023-11-10

**Authors:** Saeed Karami Mehrian, Nasser Karimi, Fatemeh Rahmani

**Affiliations:** 1 Department of Biology, Faculty of Science, Razi University, Kermanshah, Iran; 2 Department of Biology, Faculty of Science, Urmia University, Urmia, Iran

**Keywords:** Physiology, Plant sciences

## Abstract

Excessive use of pesticides against pests has contaminated agricultural crops and raised global concerns about food safety. This research investigates the alleviation effects of 24-epibrassinolide (EBL) seed priming on diazinon (DZ) pesticide toxicity. The experiment was conducted with eight groups including control, DZ, EBL (10 µM), EBL (0.1 µM), EBL (0.01 µM), EBL (10 µM) + DZ, EBL (0.1 µM) + DZ, and EBL (0.01 µM) + DZ. Plants grown with the lowest concentration of EBL (0.01 µM) exhibited an upward increase in the activity of SOD, CAT, POD, APX, GR, and GST enzymes under DZ toxicity stress. In contrast, higher concentrations of EBL showed some inhibitory effects on the activity of antioxidant enzymes. In addition, low concentrations of EBL elevated the free radical scavenging capacity (DPPH), iron-reducing antioxidant power (FRAP), photosynthesis rate (Pn), stomatal conductance (Gs) and proline, and protein contents. EBL also reduced lipid peroxidation (MDA levels) in the DZ-exposed plants, leading to membrane integrity. The favorable effects of EBL were more evident when plants were exposed to pesticides than normal growth conditions. The results indicated that EBL seed priming intensifies the antioxidant enzymes system activity, and helps maize plants against toxic effects of DZ under proper concentration.

## Introduction

The reduction in the yield and quality of crops every year forced farmers to use more pesticides to get rid of pests^1^ which has raised global concerns about food safety ^2,3^. Unaware and regardless of the side effects of pesticides, about 4.15 million tons of pesticides are used annually worldwide. Asia with more than 50% of the world’s total consumption is the top contributor, followed by the US and Brazil^4^. Regrettably, a direct relationship between many diseases including diabetes, various cancers, and of course common diseases of the present century such as autism, Parkinson’s, obesity, and hormonal abnormalities has been shown to be related to the excessive presence of pesticides in agricultural products^5,6^. More than 50 pesticides have been mentioned as the cause of hormonal disorders and their accumulation in the cells leads to an increase in metabolic syndrome and obesity^6,7^. It has been shown that overweight people with high levels of pesticides in their bodies are at higher risk of type 2 diabetes than others^8^.

Pesticides also cause adverse effects on the growth, regulation, and development of non-target plants by inducing oxidative stress and accumulation of reactive oxygen species (ROS)^9^. The negative effects of pesticides on photosynthesis, photophosphorylation, protein synthesis, and membrane lipids integrity have been reported in varieties of crops^10–12^. Diazinon (DZ) is an organophosphorus pesticide that is widely used in agriculture to control a broad spectrum of insects on vegetables, fruits, nuts, and crop plants^13^ and induces neurotoxic impacts in humans through the inhibition of acetylcholinesterase^14^.

Plant hormones alleviate abiotic stress by ameliorating the adverse effects of ROS^15–17^. Brassinosteroids (BRs) (as polyhydroxy steroids) affect seed germination, seedling growth, vascular formation, cell division, reproduction, and flower and fruit formation^18^. They play a vital role in activating antioxidant enzymes, and producing osmotic protective substances^15–17,19,20^. The hormone functions in the recovery of plants under different biotic and abiotic stress conditions such as heavy metals, salt, drought, and heat^11,15,18,19,21,22^.

The exogenous application of BRs enhances the activities of enzymes involved in the three-phased pesticide detoxification system^23,24^. In order to detoxify, in the first phase, absorbed pesticides metabolically are activated by P450 monooxygenase, peroxidase, and carboxylesterase enzymes. In the second phase, activated components are conjugated to glutathione and glucose by GST and UGT, respectively. In the third phase, soluble metabolites resulting from pesticide breaking will be stored either in vacuoles or in the apoplast^25^. Improvements in the growth and yield of crop plants and enhancement of resistance to abiotic stresses such as pesticides have already been reported as advantages of BR^22,23,26^. Seed priming with BR helps growth and establishment of seedlings under biotic and abiotic stresses and confers higher performance under adverse conditions^27^.

Little is known about cost-effective solutions to reduce pesticide residues in agricultural products. Therefore, understanding the factors influencing pesticide metabolism can help developing new detoxification methods to provide maximum crop protection which subsequently leads to minimal public health problems^23^. Based on literature review, this is the first attempt to provide conclusive evidences that EBL seed priming application alleviates DZ-induced oxidative stress via enhanced antioxidant defenses and ROS inhibition in plants.

## Results

### Seed germination and root elongation

**Figure 1:**
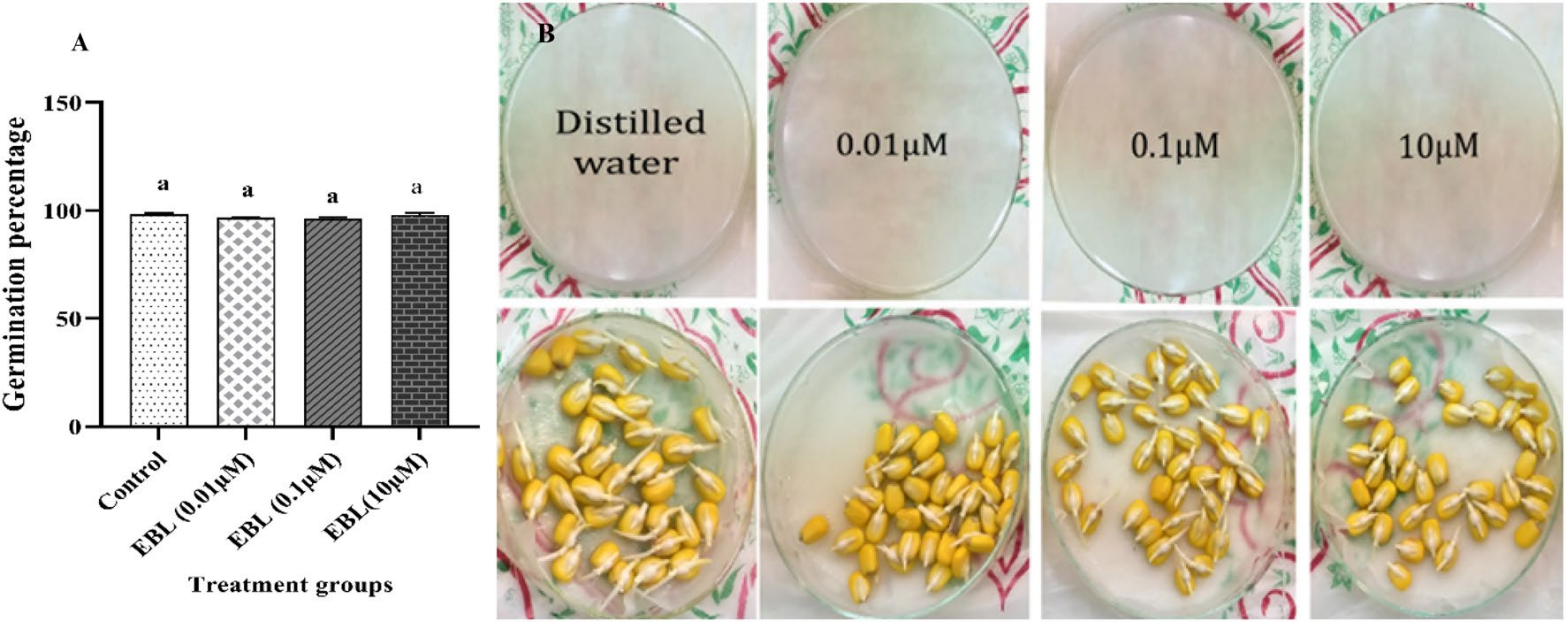
Effect of different concentrations of the EBL seed priming on the germination percentage (**A**) and seedling growth (**B**) in maize. Different letters on the bars indicate significant differences between treatments according to Turkey’s test (p < 0.05).

Different concentrations of EBL did not exert any significant impact on germination percentage, but it caused a reduction in root elongation compared to the control group (as shown in Fig. 1A,B).

### Photosynthesis

**Figure 2:**
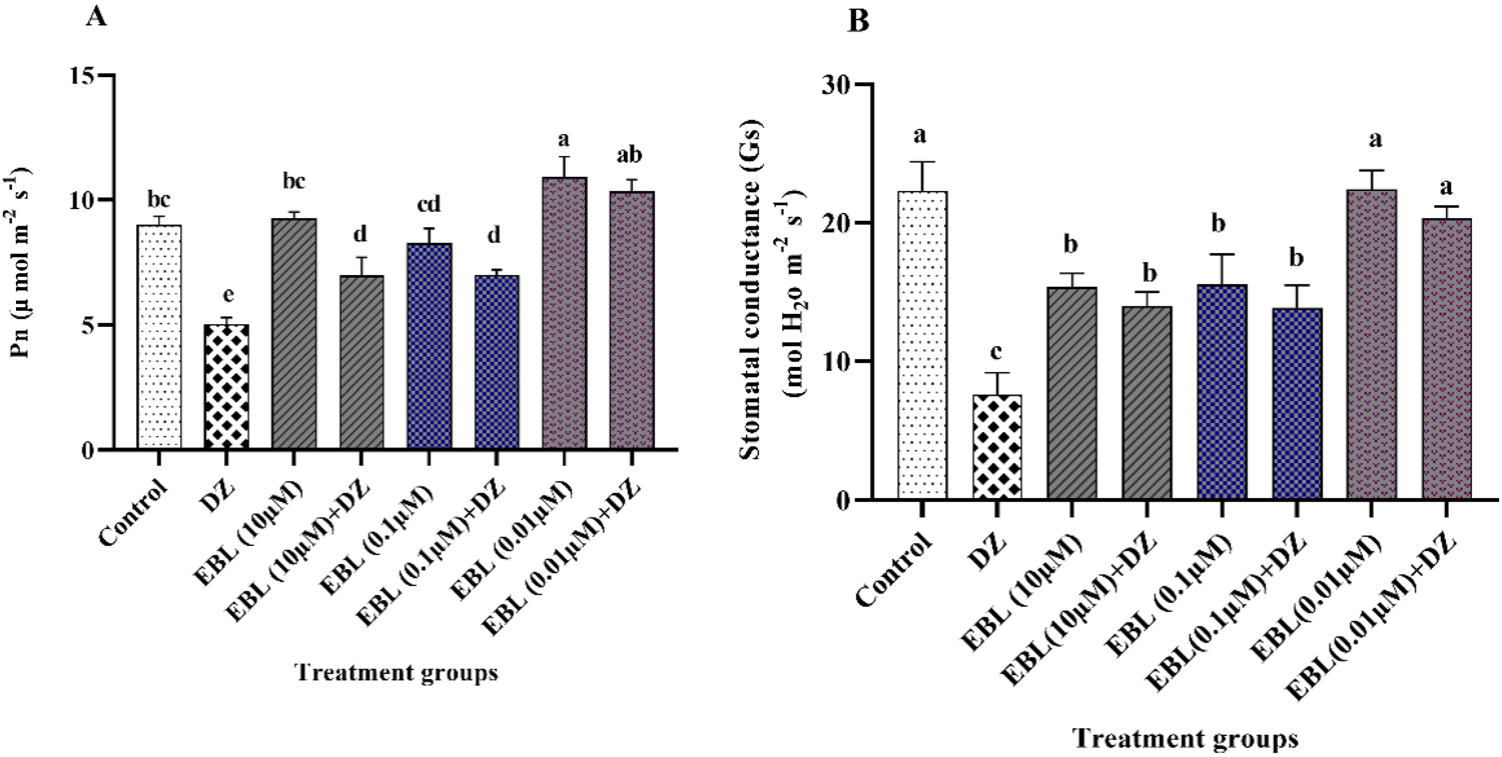
Effect of different concentrations of the EBL seed priming on the photosynthesis (Pn) and stomatal conductance rate (Gs) in the maize seedlings exposed to DZ (**A**,**B**). Different letters on the bars indicate a significant difference between treatments according to Tukey's test (p < 0.05).

A high rate of photosynthesis (Pn) was exhibited in the control and all groups treated with EBL compared to plants that received only DZ (Fig. 2A). The EBL (0.01 µM) and EBL (0.01 µM) + DZ groups displayed 118 and 106% increases in Pn compared to the DZ group. The lowest Pn rate was recorded for DZ plants.

### Stomatal conductance (Gs)

Stomatal conductance in the control and all other groups pretreated with EBL was higher than that in the DZ group. EBL (0.01 µM) and EBL (0.01 µM) + DZ groups showed 192 and 165% increase in the Gs rate, respectively, compared to the DZ group (Fig. 2B). The lowest Gs rate was for plants exposed to DZ.

### Protein content

**Figure 3:**
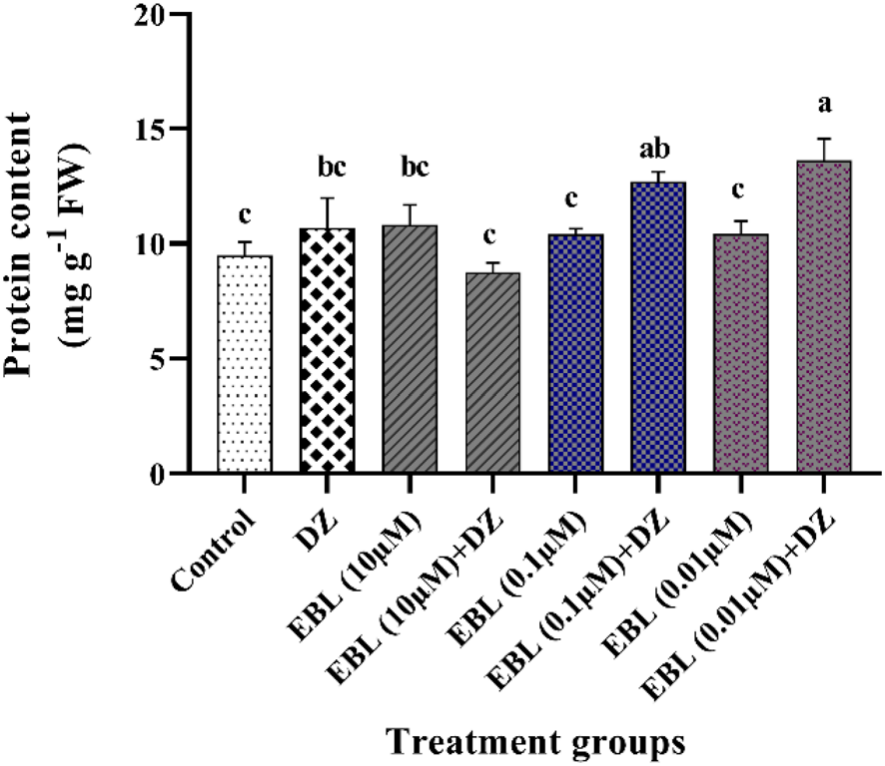
Effect of different concentrations of the EBL seed priming on the protein content in the maize seedlings exposed to DZ. Different letters on the bars indicate a significant difference between treatments according to Tukey's test (p < 0.05).

EBL seed priming enhanced protein content in the EBL (0.01 µM) + DZ treated plants (43 and 27%) compared to the control and DZ groups, respectively. In EBL (0.1 µM) + DZ treatment (33 and 18%) increases in protein content were recorded in comparison to the control and DZ groups (Fig. 3) while no significant reduction in protein content was recorded in EBL (10 µM) + DZ plants compared to the control and DZ exposed plants.

### MDA level

**Figure 4:**
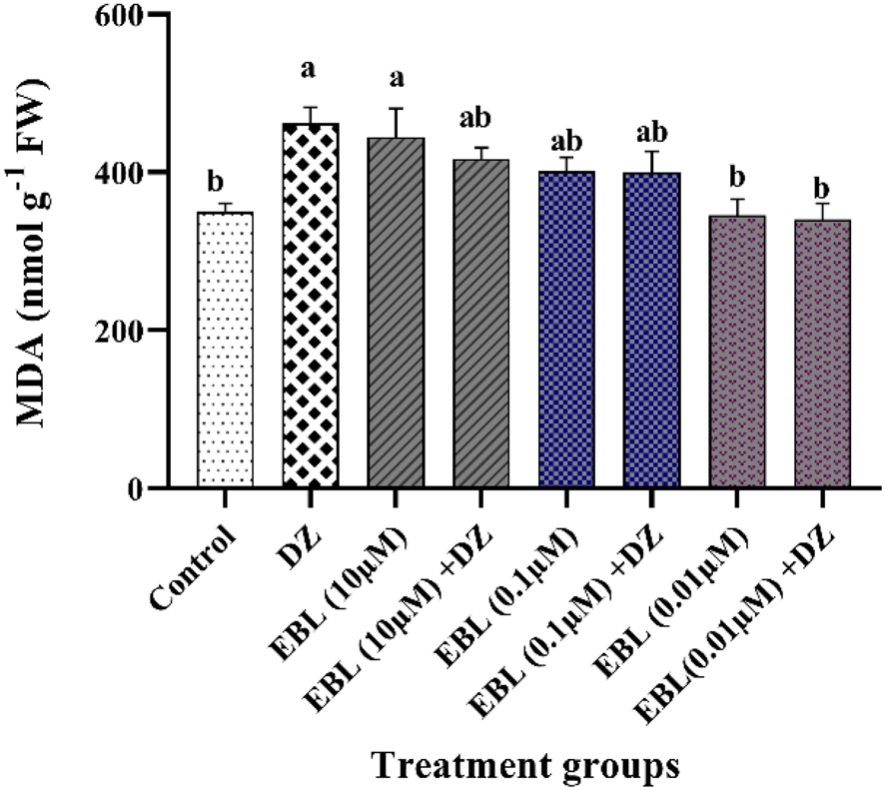
Effect of different concentrations of the EBL seed priming on MDA level in the maize seedlings exposed to DZ. Different letters on the bars indicate a significant difference between treatments according to Tukey's test (p < 0.05).

Seed priming in plants subjected to EBL (0.01 µM) + DZ reduced lipid peroxidation by 35% compared to the plants which received only DZ (Fig. 4). Higher concentrations of EBL + DZ did not significantly decrease MDA content compared to the group that received only DZ.

### Proline content

**Figure 5:**
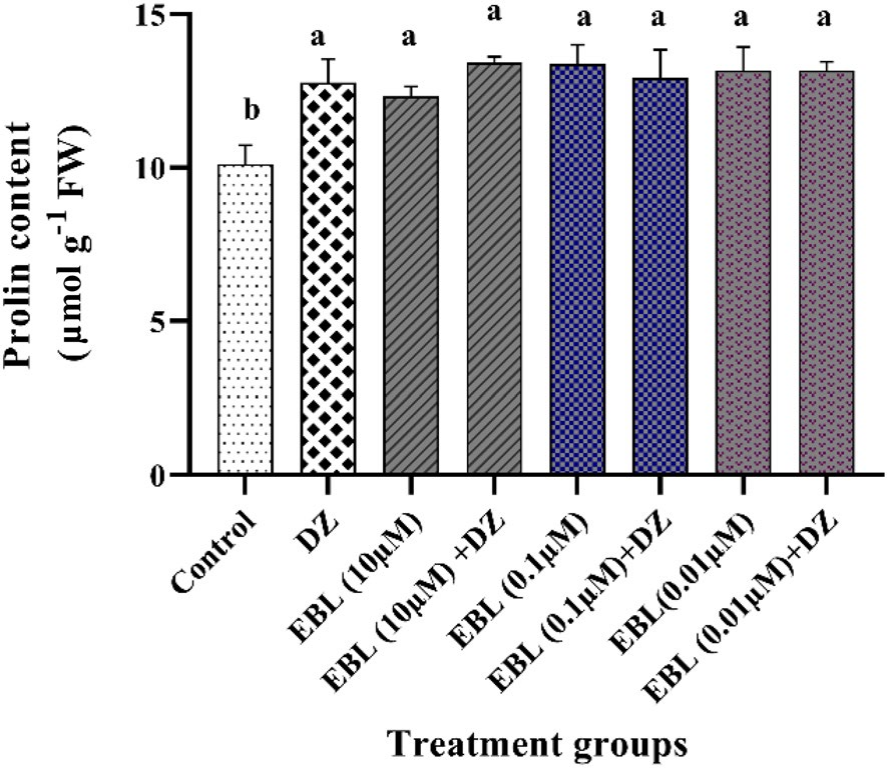
Effect of different concentrations of the EBL seed priming on the proline content in maize seedlings exposed to DZ. Different letters on the bars indicate a significant difference between treatments according to Tukey's test (p < 0.05).

Proline content was significantly increased under all treatments compared to the control group (Fig. 5), while no significant difference was observed between the EBL seed primed groups, and the DZ-treated plants.

### Antioxidant enzymes activity

**Figure 6:**
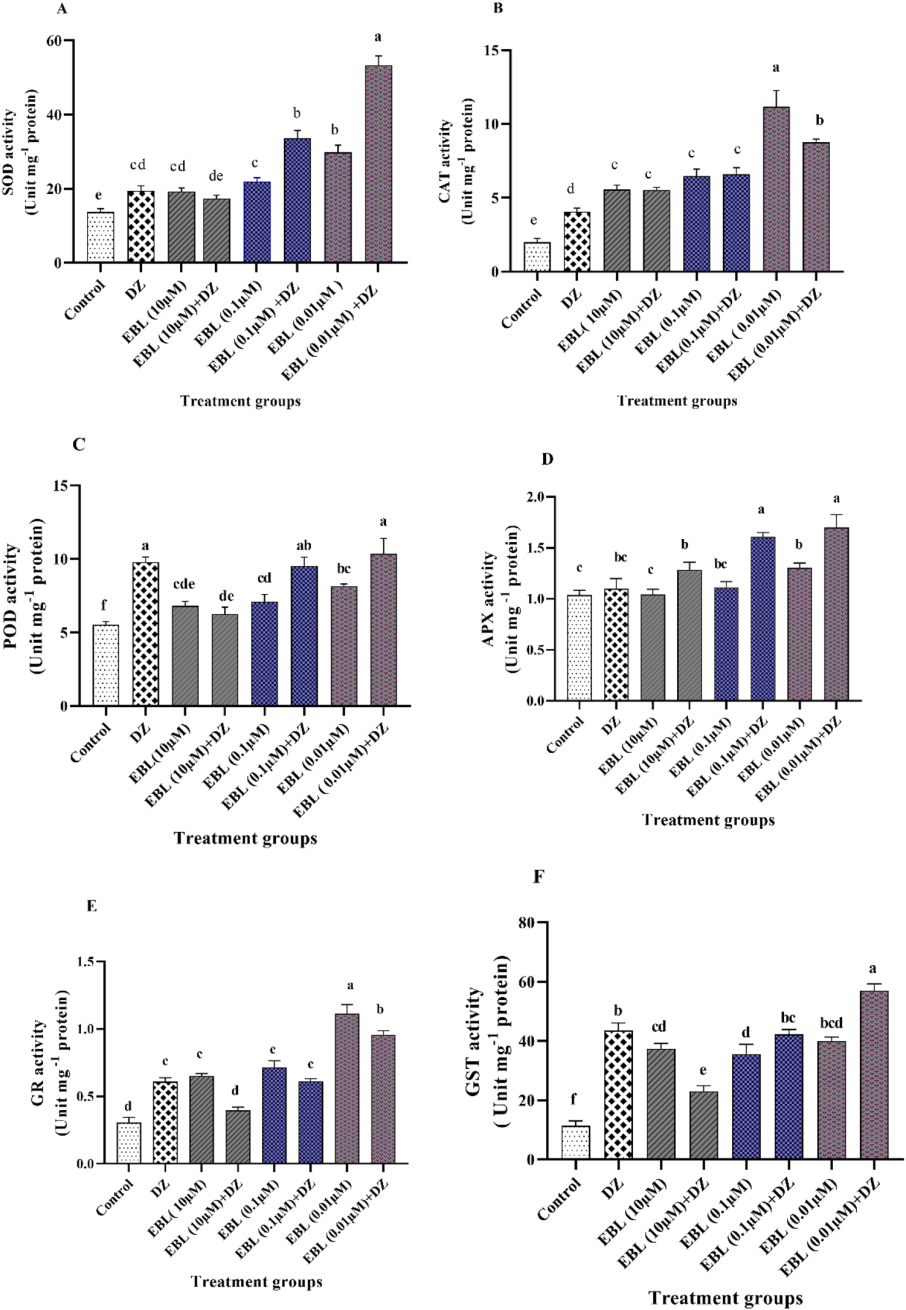
Effect of different concentrations of the EBL seed priming on the SOD (**A**), CAT (**B**), POD (**C**), APX (**D**) GR (**E**), and GST (**F**) enzymes activity in maize seedlings exposed to DZ.

All treatments except the plants treated with EBL (10 μM) + DZ showed a remarkable rise in SOD activity compared to the control group. The highest activity was recorded with an increment of 288 and 174% for EBL (0.01 µM) + DZ treated plants compared to the control and DZ groups, respectively (Fig. 6A). In general, the SOD activity increased with decreasing the EBL concentration.

Seed pre-soaking with EBL enhanced CAT activity in all treatments compared to control and DZ groups (Fig. 6B). The EBL (0.01µM) plant group with (482 and 175% increase) and the EBL (0.01 µM) + DZ treatment with (340 and 116% increase) exhibited the highest activities compared to the control and DZ groups, respectively.

The EBL (0.01 µM) + DZ, EBL (0.1 µM) + DZ, and DZ treatments showed the highest POD activity compared to the control and other treatment groups. The lowest POD activity belonged to control plants compared to the others (Fig. 6C).

A significant enhancement in the APX activity was observed in the treatments of EBL (0.01 µM) + DZ (64 and 53%) and EBL (0.1 µM) + DZ (55 and 45%) compared to the control and DZ groups, respectively (Fig. 6D).

Seed priming with the lowest EBL concentration enhanced the GR activity in EBL (0.01 µM) + DZ treatment compared to the plants that received DZ only (Fig. 6E). The maximum rise was recorded for EBL (0.01 µM) with (270 and 85%) and EBL (0.01 µM) + DZ with (216 and 58%) compared to the control and DZ groups, respectively. Exposure to EBL (10 µM) + DZ did not show any significant impact compared to control plants.

The EBL (0.01 µM) + DZ treatment exhibited an increase in the GST activity (400 and 32%) compared to the control and DZ groups (Fig. 6F). Other EBL treated groups displayed a higher GST activity compared to the control group while this elevation was not significant in the DZ treated plants.

### Free radical scavenging potential (% DPPH)

**Figure 7:**
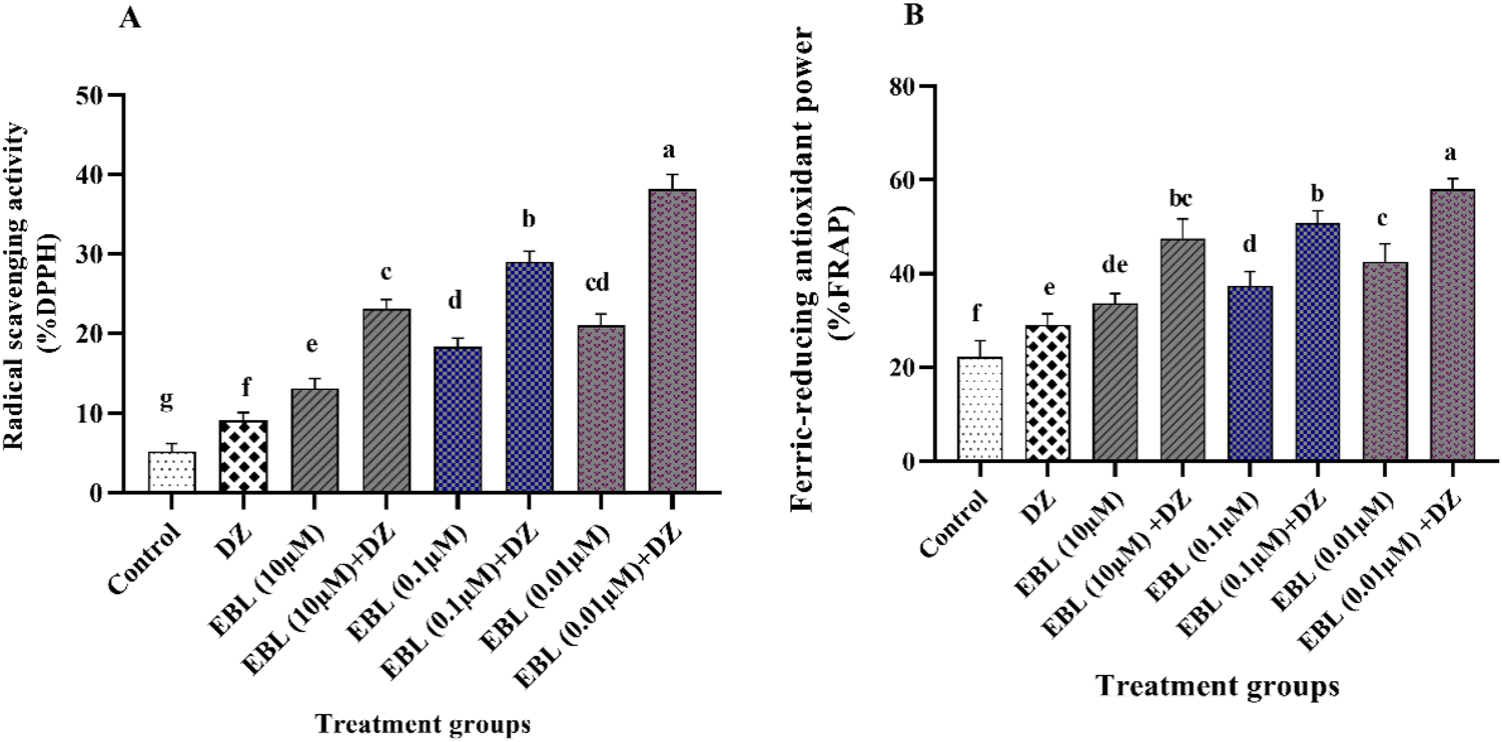
Effect of different concentrations of the EBL seed priming on the free radical scavenging potential (DPPH) (**A**) and ferric-reducing antioxidant power (FRAP) percentages in maize seedlings exposed to DZ (**B**). Different letters on the bars indicate significant differences between treatments according to Tukey's test (p < 0.05).

An upward increase in the free radical scavenging potential (DPPH) level was observed with all EBL-treated plants compared to the control and DZ groups (Fig. 7A). The DPPH radical scavenging activity was elevated with decreasing the EBL concentration. The EBL (0.01 µM) + DZ plants recorded the highest radical scavenging potential with 638 and 319% increases compared to the control and DZ groups, respectively.

### Ferric-reducing antioxidant power (FRAP)

A remarkable enhancement in FRAP value was recorded in all EBL treatments compared to the control and DZ groups (Fig. 7B). The EBL (0.01 µM) + DZ treated plants with 161 and 100% rise displayed the maximum reducing power compared to the control and DZ groups, respectively. An elevation in FRAP antioxidant capacity was evident following decrease in the EBL concentration.

## Discussion

In this research, no change was detected in the germination percentage under the EBL seed priming when compared to the control group, while a decrease in root elongation was observed in the EBL-treated seedlings (data not shown here for brevity). The stimulatory or inhibitory effects of EBL on root elongation are dependent on the concentration. High concentrations of EBL increase the expression of various IAA genes and inhibit the auxin signaling which reduces the root meristem division and prevents lateral root formation^28^. It should be noted that seeds of different species may react differently to similar EBL concentrations based on the coating thickness, pore size, and permeability^29^. Germination of *Ailanthus altissima* was promoted with 0.4 mg L^−1^ BR concentration^30^, while *Robinia pseudoacacia* and *Pinus tabulaeformis* showed the best germination percentage at 0.1 and 0.05 mg L^−1^ concentrations^31^. The favorable effect of BR on seed germination and growth is more obvious under stress and less evident under optimal conditions^32^. Even a slightly negative response to the BR treatment was found for Douglas fir under normal conditions^32^.

EBL pre-treatment significantly alleviated the destructive effect of DZ on Pn. DZ suppresses the Pn through a decrease in CO_2_ assimilation and inhibition of specific steps of the Benson–Calvin cycle and RuBisCO activity^33^. The reduction in Pn could be due to a decrease in the photochemical efficiency of PSII through suppressing the electron transport chain^33^. BR improves CO_2_ assimilation through the enhancement of the expression of related genes and the activity of enzymes involved in the photosynthesis process^16,17,23,33–35^. The EBL pretreatment increased the stomatal conductivity in the DZ-exposed plants, which could strongly affect CO_2_ assimilation and Pn. The pesticide effects on the stomatal conductance are a feedback regulation of photosynthesis^36^, which occurs due to the inhibition of CO_2_ assimilation. Mainly, BR promotes stomatal opening via inducing starch degradation by β-AMYLASE1 (BAM1) and α-AMYLASE3 enzymes in guard cells^37^. BR induces stomata opening at low concentrations and stomata closure at high concentrations^23,38,39^. A low concentration of BR activates the respiratory burst oxidase homolog and elevates H_2_O_2_ levels, which induces stomatal opening. In contrast, a high concentration of EBL causes H_2_O_2_ accumulation and decreases K^+^ uptake into stomatal guard cells, leading to the actuation of abscisic acid and stomata closure^40^.

Seed presoaking with EBL in EBL 0.01 µM + DZ experimental group, promoted soluble protein content while DZ-subjected plants showed a reduction in protein content. There are two possible reasons for the decrease in protein levels in plants under DZ stress. Firstly, increased protease activity or autophagy process that degrades the oxidized proteins under environmental stress^41^. Secondly, the unavailability of essential elements such as N, P, and S, as integral constituents in protein metabolism^42^. The toxic effect of pesticides and fungicides causes alteration in nitrogen and carbon metabolism and thereby limiting the plant's access to nutrients for growth^43^.

The 24-EBL and 28-HBL applications enhanced protein content in rice seedlings receiving Imidacloprid (IMI) and Chlorpyrifos (CPS)^11,44,45^. Seed presoaking with 0.1 µM EBL increased protein content in the leaves of the *Brassica juncea* L. plants grown in IMI contaminated soil^42^. Probably, BR has a positive effect in restoring the protein content via modulating the synthesis of polypeptides under normal and stress conditions^46^. There are pieces of evidence indicating that seed priming with BR plays a crucial role in enhancing protein content through transcriptional and translational regulations^47–49^.

Lipid peroxidation is an indicator of ROS accumulation and expressed as MDA content^50^. A dramatic rise in MDA contents for DZ and high EBL-subjected plants indicated oxidative stress, while the lowest EBL concentration application helped plants overcome this crisis through ROS scavenging^26,34^. EBL accelerated ROS scavenging and diminished lipid peroxidation through enhancing antioxidant enzymes activity in pesticides and EBL exposed rice plants^11^. A significant decrease in the MDA content of IMI-treated *Brassica juncea* L. plants presoaked with 100 nM of EBR has been previously reported^26^.

A high proline content was observed in plants imposed to EBL and DZ. Proline is considered an important osmolyte and a signal of biotic or abiotic stress conditions which its significant rise confers tolerance to stressed plants. This amino acid has a protective role against singlet oxygen and free radical-induced damage which leads to stabilization of proteins, DNA, and membranes^51–53^. Lipid peroxidation results in membrane instability, which in turn gives rise to osmotic stress. Therefore, elevation in proline content under EBL pretreatment produces an appropriate response to osmotic stress.

The activity of antioxidant enzymes (such as SOD, CAT, POD, APX, GR, and GST) in DZ group plants pretreated with EBL, showed a significant increment in the elimination of ROS. High activity of antioxidant enzymes following the application of BR could be due to the changes in the synthesis of these enzymes as a result of up-regulation in their gene expression^34^. Many antioxidant enzymes are Ca^2+^ dependent, and BRs activate calcium/calmodulin-dependent protein kinases, which can provide the basis for more activity of antioxidant pathways^54^. BZR1 and BES1 are important transcriptional factors that regulate different aspects of plant growth and development. They induce responses to various biotic and abiotic environmental stresses and are strongly regulated by exogenous BR. Moreover, overexpression of genes encoding antioxidant enzymes such as ZmSOD, ZmCAT, ZmAPX, and ZmGR are positively correlated with *ZmBZR1* and *ZmBES1* transcripts abundance^55^. Apparently, BR recognizes promotors of *BZR1* and *BES1* transcription factors and induces their phosphorylation/dephosphorylation, leading to up/down-regulation of antioxidant genes expression^55^. SOD and CAT play a critical role under stress conditions. SOD is responsible for preventing the accumulation of O2˚^−^, through the conversion of superoxide anions into hydrogen peroxide and oxygen and CAT functions in reducing the accumulation of H_2_O_2_ by converting hydrogen peroxide to water and oxygen^26^. An enhancement in POD and APX antioxidant activities in EBL-treated plants confirms the important role of these enzymes in DZ detoxification. POD is involved in the detoxification of xenobiotic compounds by oxidizing and deactivating the chemical xenobiotic^22^. APX and GR are major H_2_O_2_-scavenging enzymes via the ascorbate–glutathione (Halliwell-Asada) cycle^56,57^. APX and ascorbate–glutathione cycle play important roles in the ROS scavenging in chloroplast, cytosol, mitochondria, and peroxisomes^58^. GR is a NADPH-dependent enzyme catalyzing the reduction of GSSG to GSH and plays a vital role in the prevention of oxidative stress^23,26^. In the ascorbate–glutathione cycle, glutathione reduces the H_2_O_2_ to water by the catalytic activity of the GR enzyme^59^. An elevation in the GR and GST enzymes activity in the EBL-treated plants was observed in this study. The increase in the GR activity may be attributed to a higher level of GSH and the formation of the GS-conjugation to pesticides, which leads to GST enzyme transporting the GSH-conjugated residues into the apoplast or vacuole^60^. GST causes the formation of less reactive and non-toxic soluble water components and more polar glutathione S-conjugates in plants by detoxification of xenobiotic compounds through covalently linking glutathione to hydrophobic substrates^61,62^. BR induces pesticide detoxification in plants by increasing the GSH content, POD, GR, and GST enzymes activity^22,63^. Interestingly, the total antioxidant status of the EBL-presoaked maize seedlings exposed to DZ was found to be higher than the EBL- or DZ-alone applications.

In this research, EBL seed priming strongly enhanced the DPPH and FRAP antioxidant capacities, indicating the positive role of EBL in the ROS elimination and prevention of lipid peroxidation. The DPPH and FRAP assays are two sets of frequently utilized approaches using electrons or radical scavenging to evaluate the ability of compounds as free-radical scavengers and hydrogen suppliers for determining the free-radical scavenging potential and limiting lipid oxidation^64^. EBR application to *Raphanus sativus* seedlings increased the levels of DPPH radical scavenging and FRAP antioxidant capacity under Cu and Cr stresses^65^. Further studies showed that EBL, Bik, and brazide applications enhanced the ROS scavenging through the elevation of antioxidant activity^35^.

## Conclusion

The results demonstrate that the EBL treatment plays an important role in the metabolism and detoxification of pesticides upon appropriate concentrations, which can be an eco-friendly strategy to promote the tolerance of plants to pesticides. EBL elevates the activity of antioxidant enzymes and enhances the contents of osmotic adjustment substances leading to a decline of lipid peroxidation. It should be noted that the desired impact of EBL varies depending on the concentration and plant species, and the positive influences are more evident when facing adverse conditions. Probably, EBL changes the expression of antioxidant genes by targeting the promoters of *BZR1* and *BES1* genes and altering their phosphorylation patterns. This results in an increase in the antioxidant enzymes’ activity and prevention of O_2_˚^−^ accumulation (through conversion of superoxide anions and reduction in the accumulation of H_2_O_2_ by converting to water and oxygen and enhancement of ascorbate–glutathione cycle) which leads to more conjugation of glutathione into pesticide residues and facilitates the transfer of pesticides residues into the vacuole or outside the cell. Finally, a proper concentration of EBL in the seed priming mode could act as an effective plant regulator to minimize the adverse effects of DZ in maize plants.

## Materials and methods

### Plant growth and treatment

Uniform-size seeds of maize (*Zea mays* 704 single cross) were purchased from the Urmia agricultural research institute of Iran, and surface sterilized by sodium hypochlorite 10% for 10 min and rinsed with distilled water three times; seeds were randomly divided into eight groups, control, DZ, EBL (10 µM), EBL (0.1 µM), EBL (0.01 µM), EBL (10 µM) + DZ, EBL (0.1 µM) + DZ and 0.01 µM EBL + DZ. For seed priming, the control and DZ seeds were soaked in distilled water (1µL of ethanol was added to the water because we dissolved the EBL in ethanol), and other groups soaked in different EBL concentrations (obtained from the Sigma-Aldrich CAS Number: 78821-43-9) for 24 h. Soaked seeds were washed with distilled water three times and transferred into the plastic pots containing soil (three replications for each treatment). The climate chamber condition was at a temperature of 28 °C/25 °C ± 2°C Day/night; photoperiod, 16 h; light intensity, 420 µmol m^−2^ s^−1^; relative humidity, 70%. When the fourth leaves of seedlings were fully expanded (30 days old plants), DZ 60% EC was applied to plants by foliar spray using a handheld sprayer until run-off. For control groups, water was sprayed. Five days after DZ treatments plants were harvested and all plants in a pot were powdered with liquid nitrogen considered as a repeat, and kept in a -80°C refrigerator for future analyses.

### Seed germination experiment

Seeds were sterilized and randomly divided into four groups, with three Petri dishes for every treatment. Petri dishes were floored with Whatman No. 1 filter paper. In each Petri dish, 50 seeds were placed and treated with 10 ml of distilled water for the control, and EBL for EBL (10 µM), EBL (0.1 µM), and EBL (0.01 µM) groups. Petri dishes were shaken several times throughout 1 h to ensure that all the seeds were thoroughly in contact with the solutions. Then kept in a growth chamber in a dark place till germination. The germination was recorded daily and the germination percentage was recorded after 4 days of treatment.

### Photosynthesis and stomatal conductance

Photosynthetic rate (Pn) and stomatal conductance (Gs) were determined by a portable photosynthesis system (HCM 1000, WALZ).

Measurement of Pn was conducted on the fourth fully expanded leaves in the period 10:00–12:00 AM. The photosynthetic photon flux density (PPFD), air relative humidity, CO_2_ concentration, and air temperature were maintained at 500 µmol m^−2^ s^−1^, 80%, 348 µmol mol^−1^, and 23–29 °C, respectively.

### Protein, MDA and proline content

Soluble protein and free proline contents were determined following the methods by Bradford^66^ and Senthilkumar et al.^67^ respectively.

The malondialdehyde (MDA) content was determined following the method by Hodges et al.^50^ with some modifications. Two-tenths of a gram of a sample was homogenized in 5 mL of 1% trichloroacetic acid at 4 °C and then the mixture was centrifuged at 8000×*g* for 10 min at 4 °C. One ml of supernatant was mixed with 4 mL of 20% TCA containing 0.5% 2-thiobarbituric acid. The samples were incubated at 95 °C for 30 min and then the reaction was stopped in the ice bath. For the second time, the samples were centrifuged at 8000×*g *for 5 min and the absorbance of the supernatant was measured at 600 and 532 nm.

### Determination of antioxidant enzymes’ activity, DPPH and FRAP assay

The Superoxide dismutase (SOD; EC 1.15.1.1) enzyme activity was measured according to the method of Beauchamp and Fridovich^68^ with some modifications. The reaction mixture comprised 50 mM potassium phosphate buffer (pH 7.8), 33 µM NBT, 66 mM EDTA, 33 µM Riboflavin, and 10 mM L-Methionine. The absorbance was monitored at 560 nm.

The Catalase (CAT, EC1.11.1.6) activity was determined according to Aebi^69^. The reaction mixture contained 100 mM phosphate buffer (pH 7.0), enzyme extract, and 200 mM H_2_O_2_ solution. The activity was recorded by following the decrease in absorbance at 240 nm for 1 min.

Peroxidase (POD, EC1.11.1.7) activity was evaluated according to Fielding and Hall^70^. The reaction mixture contained 10 mM H_2_O_2_, 50 mM phosphate buffer (pH 7), and 9 mM guaiacol, and the enzyme extract was mixed well in the cuvette and the activity was estimated by the increase in guaiacol absorbance at 470 nm.

Glutathione S-transferase (GST; EC 2.5.1.18) activity was measured by the following method given by Habig and Jakoby^71^. The reaction mixture comprised of 100 mM phosphate buffer (pH 6.5),1 mM EDTA, GSH 75 mM, CDNB 30 mM in Ethanol 95%, and 100µL of GST (enzyme extract). The changes in the reduction at 340 nm were monitored for 5min.

Glutathione reductase (GR; EC 1.6.4.2) activity was assessed according to Foyer and Halliwell^72^. The reaction mixture contained phosphate buffer 50 mM, 2.5 mM MgCl_2_, 20 nM NADPH, 0.5 mM GSSG, and enzyme extract. The decrease at 340nm in 1 min was monitored.

The activity of Ascorbate peroxidase (APX; EC 1.11.1.11) was assayed following the method of Nakano and Asada^73^. The reaction comprised of 50 mM phosphate buffer (pH 7.0), 10 mM ascorbic acid, 100 nM Na_2_EDTA, and 1 mM H_2_O_2_. The changes in absorbance were recorded at 290 nm. All the enzyme activities were expressed as mg^−1^ protein min^−1^.

Total antioxidant status was evaluated by performing the 1,1-diphenyl-2-picrylhydrazyl (DPPH) as described by Choudhary et al.^74^. The reaction mixture containing 30 mM DPPH, absolute ethanol, and enzyme extract was shaken vigorously and kept for 30 min in a dark place at room temperature. The change in absorbance was recorded at 515 nm. A blank tube containing DPPH radical without enzyme extract was also measured. The radical scavenging capacity was calculated using the following equation. DPPH Scavenged (%) = ((AB –AA)/AB) × 100, where AB is the absorbance of blank at t = 0 min, and AA is the absorbance of the antioxidant at t = 30 min.

The Ferric Reducing Antioxidant Power (FRAP) reducing power was performed as the procedure of Benzie and Strain^75^. Three ml reaction mixture containing 300 mM acetate buffer, 200 µM TPTZ in 40 mM HCl, 20 mM FeCl_3_ 6H_2_O, distilled water, and the methanolic extract were incubated for 30 min. After that time when the (Fe^3+^ TPTZ) complex was reduced to (Fe^2+^) form, the changes in absorbance at 593 nm were monitored for the enzymes and blank tubes.

### Statistical Analysis

One-way analysis of variance (ANOVA) followed by Tukey HSD tests was employed to examine the differences between various treatments at 5% probability by SPSS software version 22. All the graphs were designed by Graph Pad Prism 9.

### Approval for plant experiments

Experimental research has been complied with institutional, national, and international guidelines and legislation.

## Data Availability

The datasets generated during and/or analyzed during the current study are available from the corresponding author on reasonable request.
